# Direct Oral Anticoagulant-Related Bleeding in Atrial Fibrillation Patients Leads to *ADAMTS7* Promoter Demethylation

**DOI:** 10.3390/genes16060698

**Published:** 2025-06-09

**Authors:** Georgia Ragia, Thomas Thomopoulos, Myria Pallikarou, Natalia Atzemian, Anthi Maslarinou, Georgios Chalikias, Athanasios Trikas, Dimitrios N. Tziakas, Vangelis G. Manolopoulos

**Affiliations:** 1 Laboratory of Pharmacology, Medical School, Democritus University of Thrace, Dragana Campus, 68100 Alexandroupolis, Greece; mpallika@med.duth.gr (M.P.); anthimas@hotmail.gr (A.M.); 2 Individualised Medicine & Pharmacological Research Solutions (IMPReS) Center, Dragana Campus, 68100 Alexandroupolis, Greece; 3 Department of Cardiology, “Elpis” General Hospital of Athens, 11522 Athens, Greece; thomopoulosthomas86@gmail.com; 4 Cardiology Department, Medical School, Democritus University of Thrace, Dragana Campus, 68100 Alexandroupolis, Greece; gchaliki@med.duth.gr (G.C.); dtziakas@med.duth.gr (D.N.T.); 5 Department of Cardiology, Evaggelismos Hospital, 10676 Athens, Greece; atrikas@otenet.gr; 6 Clinical Pharmacology Unit, Academic General Hospital of Alexandroupolis, Dragana Campus, 68100 Alexandroupolis, Greece

**Keywords:** miR-CRAFT study, direct oral anticoagulants, rivaroxaban, apixaban, dabigatran, *ADAMTS7*, methylation, epigenetics

## Abstract

Background/Objectives: Among other substrates, the a disintegrin and metalloproteinase with thrombospondin motifs 7 (*ADAMTS7*) protease degrades thrombospondin-5 (the cartilage oligomeric protein, COMP), thrombospondin-1 (TSP-1) and the tissue inhibitor of metalloproteinases-1 (TIMP-1) indicating a potential role of *ADAMTS7* expression on coagulation cascade, tissue remodeling and wound healing. We analyzed the potential effect of direct oral anticoagulant (DOAC) treatment on *ADAMTS7* promoter methylation and followed it over time to assess whether DOACs epigenetically modulate *ADAMTS7* and induce pathways associated with coagulation or endothelium repair machinery. Methods: Eighty-four DOAC-treated atrial fibrillation (AF) patients followed-up from baseline (t0) to 7 days (t1, *n* = 70) and 28 days of treatment (t2, *n* = 62) and 19 non-AF controls were included in the study. Genomic DNA was extracted from blood at all timepoints and was bisulfite-converted prior to methylation analysis. *ADAMTS7* promoter DNA methylation was analyzed with MIP-qMSP-PCR. Results: A total of 16 minor bleeding events occurred. The baseline percentage of *ADAMTS7* methylation did not differ between AF patients and controls (15.8% vs. 16.1%, *p* = 0.908). In the patient cohort, DOAC therapy marginally decreased *ADAMTS7* methylation from t0 to t2 (15.2% vs. 14.0%, *p* = 0.044). This *ADAMTS7* demethylation from t0 to t2 was statistically significant only in patients experiencing bleeding (17.1%. vs. 13.4%, *p* = 0.010 in bleedings, 14.5% vs. 14.2%, *p* = 0.561 in non-bleedings). No other differences were observed. Conclusions: *ADAMTS7* is demethylated during DOAC-related bleedings, a mechanism potentially leading to COMP degradation and thus thrombin-induced platelet aggregation, as well as the induction of endothelium repair through different *ADAMTS7*-dependent pathways.

## 1. Introduction

Pharmacoepigenetics, the study of drug-induced epigenetic modifications, remains an emerging and underexplored field, particularly in the context of cardiovascular therapies [1]. While DNA methylation has been recognized as a key mechanism regulating gene expression, little is known about whether widely used cardiovascular drugs, such as direct oral anticoagulants (DOACs), can influence epigenetic marks *in vivo*.

The a disintegrin and metalloproteinase with thrombospondin motifs (ADAMTS) family consists of 19 metzincin metalloproteinases, which are secreted proteins that degrade the extracellular matrix (ECM) [2]. Due to their role in the regulation of the structure and function of extracellular proteins in the extracellular matrix and blood, ADAMTS proteases have attracted attention as possible participants in disease pathogenesis [3]. In the cardiovascular system, *ADAMTS7* shows strong association with coronary artery disease and atherosclerosis promotion [4]. *ADAMTS7* substrates are not yet well understood [5,6]; however, *ADAMTS7* has been defined as thrombospondin-5 (the cartilage oligomeric protein, COMP) protease [7], while additionally it cleaves thrombospondin-1 (TSP-1) [8] and the tissue inhibitor of metalloproteinases-1 (TIMP-1) [9]. COMP is a natural thrombin activity inhibitor [10] indicating that, through its substrate, *ADAMTS7* may also have a role in coagulation. On the other hand, TSP-1 is a known endogenous angiogenesis inhibitor [11], whereas TIMP-1 regulates tissue remodeling and wound healing through matrix metalloproteinase (MMP) inhibition [12].

*ADAMTS7* gene expression is regulated both transcriptionally and post-transcriptionally. Proinflammatory element binding sites can be found in the promoter region, indicating that transcription factors regulate *ADAMTS7* transcription in response to inflammatory stimuli [13]. Additionally, microRNAs suppress *ADAMTS7* translation, adding to an overall complex *ADAMTS7* regulation [14,15,16]. More recently, data has been published on the effect of *ADAMTS7* promoter methylation on *ADAMTS7* expression, and increased gene expression in hypomethylated *ADAMTS7* has been reported [17]. In this study, the authors have additionally shown that demethylation induced by 5’-Aza directly restored *ADAMTS7* expression in trophoblast cells [17]. To date, it is largely unknown whether drugs commonly used in pharmacotherapy could have an effect on *ADAMTS7* promoter methylation, epigenetically altering *ADAMTS7* expression and, thus, inducing *ADAMTS7*-related pathways.

Given the role of *ADAMTS7* in the cardiovascular system and more specifically on the coagulation pathway, we have herein hypothesized that therapy with DOACs may modify *ADAMTS7* promoter methylation, leading to a broader regulation of the coagulation cascade through the inhibition of COMP, or induce different *ADAMTS7*-substrate-dependent mechanisms as a response to DOAC-related bleeding. The primary aim of our study is to follow the effect of DOAC (rivaroxaban, apixaban or dabigatran) treatment over time on *ADAMTS7* promoter methylation in newly diagnosed non-valvular atrial fibrillation (AF) patients, both in pooled analysis as well as stratified in bleeding cases and controls. Secondarily, we have studied the effect of other factors, including AF incidence, sex and common comorbidities, on *ADAMTS7* methylation patterns.

## 2. Materials and Methods

### 2.1. Study Population

**Figure 1:**
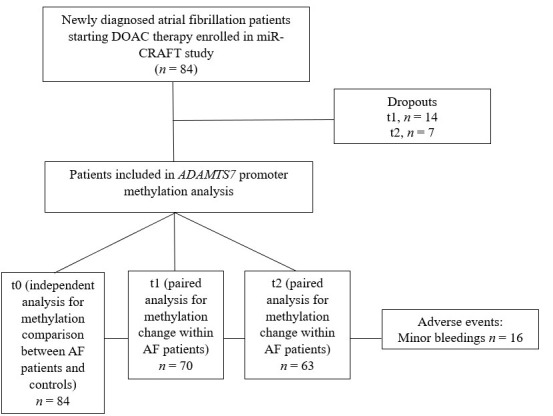
Analytic flowchart of miR-CRAFT patients included in *ADAMTS7* promoter methylation analysis.

A total of 84 atrial fibrillation (AF) patients, participants of a miR-CRAFT study [18], and 19 non-AF controls were included in the *ADAMTS7* promoter methylation analysis. Twenty-one patients did not return for follow-up visits at 7 days (*n* = 14) or 28 days (*n* = 7). Finally, 70 patients were included in the paired analysis for methylation change from baseline to 7 days of treatment, and 63 patients were included in the analysis from baseline or 7-day treatment to 28-day therapy (See Figure 1).

The miR-CRAFT study design has been described earlier [18,19]. In brief, the miR-CRAFT population consisted of newly diagnosed AF patients initiating DOAC treatment with rivaroxaban, apixaban or dabigatran. Epigenetic modifications, such as DNA methylation and microRNA expression, are analyzed at three different timepoints: at baseline (t0) and after seven (t1) and twenty-eight days (t2) of DOAC treatment. During follow-up visits, potential adverse event incidences, including both major or clinical insignificant bleeding events, were recorded. All subjects participated after being informed about the study and giving written consent. The study protocol was approved by the Ethical Committee of Athens General Hospital “Elpis” (approval ΕΣ 23/14 April 2019) and of the Academic General Hospital of Alexandroupolis (approval ΕΣ 3/3 February 2022).

### 2.2. Genomic DNA Purification and Bisulfite Conversion

From all participants, approximately 3 mL blood was collected in ethylenediaminetetraacetic acid (EDTA) tubes at baseline for the control group and at t0, t1 and t2 for patients for DNA purification. Genomic DNA was extracted within 72 h from peripheral whole blood using MagCore Automated Nucleic Acid Extractor (RBC Bioscience, New Taipei City, Taiwan), according to the instructions of the manufacturer, and was stored at −20 °C until use. DNA purity and quantity were assessed with a UV–Vis Spectrophotometer Q5000 (Quawell, San Jose, CA, USA). Sodium bisulfite (SB) conversion of genomic DNA (300 ng input) was performed using the EZ DNA Methylation-GoldTM kit (Zymo Research Corporation, Irvine, CA, USA), according to the manufacturer’s protocol. The converted DNA was stored at −80 °C until use. SB modification resulted in the conversion of un-methylated cytosine to thymine, whereas the methylated cytosine remained unaltered.

### 2.3. Analysis of DNA Methylation

For the methylation analysis of the *ADAMTS7* gene promoter, an initial methylation independent polymerase chain reaction (PCR) (MIP) was performed, followed by a nested quantitative methylation–PCR (qMSP).

The MIP primers were designed based on sequence NC_000015.10 (Homo sapiens chromosome 15, GRCh38.p14 Primary Assembly, nucleotides 78811645–78812344). The following primers were used: MIP forward primer, 5’-AGATTAATTAAGGGGAGGAAGTTGAT-3’ (78812375–78812401); MIP reverse primer 5’-AAACAAACCTAAACCCTCCCTAA-3’ (78812773–78812796). Each MIP PCR reaction (final reaction volume 25 μL) contained 2.5 μL of 10× TaqNovaHS Buffer, 1.5 μL of 25 mM MgCl_2_, 0.5 μL of 10 mM dNTPs, 0.15 μL of MIP forward and MIP reverse (100 μM concentration) primer, 0.5 μL of 5 U/μL TaqNovaHS DNA polymerase (Qiagen, Lancashire, United Kingdom), and 1.5 μL (approximately 45 ng) of SB-converted DNA. PCR conditions were as follows: 95 °C for 2 min, 40 cycles of 95 °C for 30 s, 57 °C for 45 s, and 72 °C for 1 min (PCR stage), followed by a final extension stage at 72 °C for 20 min. The MIP product was 420 bp.

For the qMSP, two sets of PCR primers, one for the unmethylated (USP forward: 5’-GGTTGGGGTTATGAAGGATAGAT-3’ and USP reverse: 5’-AACAAACAACAATTACCCAC-3’, PCR product 251 bp) and one for the fully methylated (MSP forward: 5’-GGGGTTACGAAGGATAGAC-3’ and MSP reverse: 5’-AACAAACAACGATTACCCG-3’, PCR product 249 bp) sequence were used, as described by Zhang et al. [17], covering a total of 4 CpG cites.

Each qMSP-PCR reaction (final reaction volume 10 μL) contained 5 μL of 2× Kapa SYBR mix (Kapa Biosystems, London, United Kingdom), 0.08 μL of MSP forward and MSP reverse primer or 0.08 μL of USP forward and USP reverse primer (all diluted to 50μM), 0.2 μL of ROX low, and 1 μL of MIP PCR product diluted to 0.5 ng/μL based on quantitative accuracy assay for concentrations 1 ng, 0.5 ng, 0.1 ng and 0.01 ng for 100% and 0% methylated MIP products.

qMSP-PCR reactions were performed in a 96-well plate on a QuantStudio™ 12 K Flex Real-Time PCR System (ThermoFisher Scientific, Waltham, MA, USA) with the following cycling parameters: 95 °C for 3 min, 30 cycles of 95 °C for 15 s, 58 °C for 50 s (PCR stage). At the end of the reaction, a melting curve analysis was performed by increasing the temperature from 55 °C to 95 °C at a rate of 1 °C per second to generate the dissociation curve. The correct length of the PCR products was verified by agarose gel electrophoresis.

Specificity and cross reactivity of the methylated and unmethylated *ADAMTS7* primers were evaluated by using a Human Methylated & Non-methylated DNA Set (Zymo Research Corporation, Irvine, CA, USA) as fully methylated (100% methylated) and non-methylated (0% methylated) MSP positive and negative controls, respectively.

To assess the sensitivity and efficiency of the *ADAMTS7* qMSP method, MIP PCR products of 100% and 0% methylated SB-converted DNA were diluted 100% to 80%, 60%, 40% and 20%. These dilutions were used as negative and positive control samples in every qMSP run. To avoid bias, methylated and unmethylated MSP was run in the same plate for each sample.

### 2.4. Statistics

The percentage of *ADAMTS7* methylation in samples was estimated using the following formula according to Lu et al. [20]:

$$\mathrm{Methylated}\;\mathrm{ADAMTS}7\;(\%)=\frac{1}{1+2^{-\Delta Ct}}\times 100\%$$

where ΔCt = Ct*_Unmethylated_* − Ct*_Methylated_*

Distribution of continuous variables was assessed with the Shapiro-Wilk test of normality. Normally distributed variables are expressed as mean ± standard deviation (SD) and were compared within two or more groups by the appropriate parametric tests (independent or paired *t*-test, one-way ANOVA). Skewed data are expressed as the median (25th, 75th percentiles) and were compared within two or more groups by the appropriate non-parametric tests (Mann–Whitney test or Kruskal–Wallis test). Nominalvariables are presented as absolute number and percentage value. s.Comparisons for categorical data between two groups were conducted using an χ^2^ test. To estimate the risk of bleeding associated with *ADAMTS7* promoter demethylation (t0-t2 methylation difference), the odds ratio (OR) was calculated using regression analysis after adjustment for age, gender, hypertension, DOAC dose and renal function. To estimate the likelihood of bleeding leading to *ADAMTS7* demethylation, beta coefficient (β) with 95% C.I.s was calculated with a multivariable linear regression analysis with *ADAMTS7* demethylation as a dependent variable and bleeding, age, gender, hypertension, DOAC dose and renal function as independent variables. Post hoc power calculation with a preset level of significance (*p* = 0.05) was performed. A *p* value less than 0.05 was considered statistically significant. Analyses were carried out with the use of the SPSS software package (version 27.0 for Windows; SPSS Inc., Chicago, IL, USA).

## 3. Results

### 3.1. MIP and qMSP Validation

The MIP PCR reaction yielded a PCR product only when BS-converted DNA was used as the template, whereas no MIP product was observed for unconverted gDNA. In nested-qMSP, the 100% methylated template yielded the PCR product only when the methylated pair of primers was used (product length 249 bp), while the non-methylated template yielded the PCR product only when the un-methylated pair of primers was used (product length 215 bp). The efficiency of the assay was 104%.

### 3.2. Population Characteristics

Patients’ demographic, biochemical and clinical characteristics are presented in Table 1. The cohort population consisted of 84 AF patients (46.4% male) of mean age 69 years (± 12). The median CHA_2_DS_2_-VASc score and HAS-BLED score were 3 (25%, 75% percentiles, 2, 4) and 0 (25%, 75% percentiles 0, 1), respectively, whereas 16 patients (27.6%) had moderately or severely decreased renal function based on Glomerular Filtration Rate (GFR). Liver function was assessed by a serum glutamic-oxaloacetic transaminase (SGOT) and serum glutamic pyruvic transaminase (SGPT) test; nine patients had increased SGOT levels (>40 U/L) and eight patients had increased SGPT levels (>56 U/L), whereas among them, three patients presented with increased SGOT and SGPT levels.

Patients were treated with rivaroxaban (*n* = 28), apixaban (*n* = 36) or dabigatran (*n* = 20). Patient characteristics did not differ among different drugs or between factor Xa inhibitors and thrombin inhibitor. No major bleeding or thrombotic events were recorded. A total of 16 minor bleeding events occurred. An increased prevalence of hypertension and dyslipidemia were present in bleeding cases. No other differences, including HAS-BLED score and antiplatelet, salicylate or nonsteroidal anti-inflammatory drug (NSAID) use, were noticed in patient characteristics between bleeding cases and non-bleeding controls (Table 1).

### 3.3. ADAMTS7 Promoter Methylation

#### 3.3.1. *ADAMTS7* Promoter Methylation in AF Patients and Control Subjects

**Figure 2:**
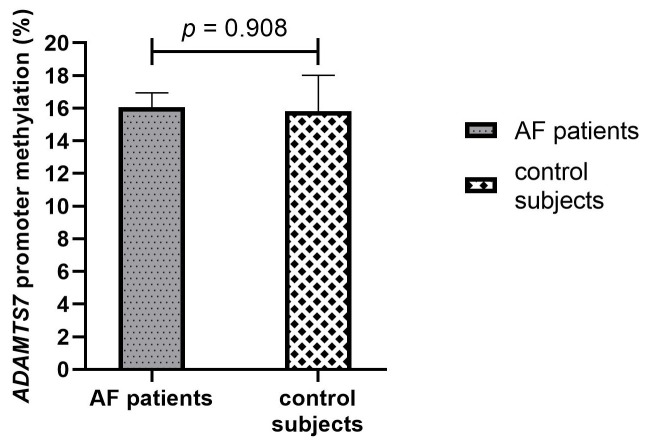
Baseline methylation percentage of the *ADAMTS7* promoter in AF patients and control subjects. AF, Atrial fibrillation.

In the total population, the *ADAMTS7* promoter was partially methylated with a mean percentage of methylation of 16.0%. The methylation pattern of the *ADAMTS7* gene promoter region did not differ at baseline between AF patients and control subjects (16.1% vs. 15.8%, *p* = 0.908), Figure 2.

#### 3.3.2. *ADAMTS7* Promoter Methylation in DOAC-Treated AF Patients

**Figure 3:**
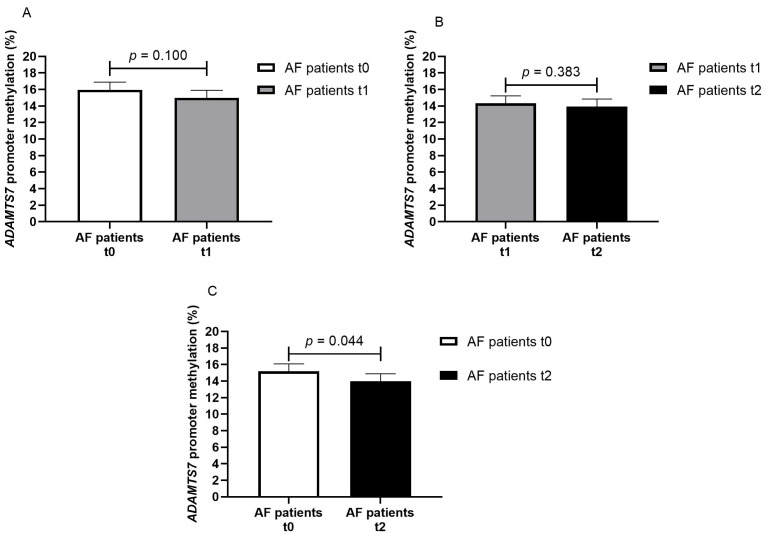
Methylation percentage of the *ADAMTS7* promoter in AF patients at different timepoints ((**A**) AF patients at baseline—t0 and on seven days of DOAC therapy—t1, paired data for 70 patients; (**B**) AF patients on seven days—t1 and on twenty-eight days of DOAC therapy—t2, paired data for 63 patients; (**C**) AF patients at baseline—t0 and on twenty-eight days of DOAC therapy—t2, paired data for 63 patients).

In the patient cohort, we analyzed the change in *ADAMTS7* promoter methylation from baseline (t0) to 7 days (t1) (paired data for 70 patients) and 28 days (t2) (paired data for 63 patients) of DOAC treatment. No difference was present from t0 to t1 (15.9% vs. 15.0%, *p* = 0.100) and from t1 to t2 (14.3% vs. 13.9%, *p* = 0.383) (Figure 3A,B). *ADAMTS7* promoter methylation was marginally different in DOAC-treated AF patients from baseline to 28 days of treatment (15.2% vs. 13.9%, *p* = 0.044), Figure 3C.

When the change in *ADAMTS7* promoter methylation was analyzed in patients stratified per drug category (factor Xa inhibitors and thrombin inhibitor), a difference was present only in factor Xa inhibitor-treated patients (mean difference −1.66% from t0 to t1, *p* = 0.016, paired data for 52 patients; mean difference −1.44% from t0 to t2, *p* = 0.041, paired data for 49 patients).

#### 3.3.3. *ADAMTS7* Promoter Methylation in Bleeding Cases and Non-Bleeding Controls

**Figure 4:**
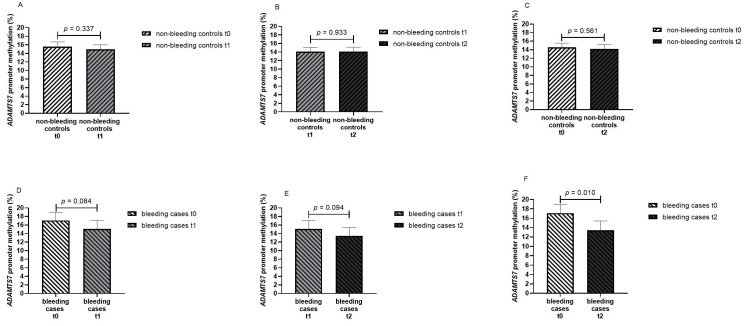
Methylation percentage of the *ADAMTS7* promoter in AF patients at different timepoints stratified by non-bleeding controls and bleeding cases ((**A**) non-bleeding controls at baseline—t0 and on seven days of DOAC therapy—t1, paired data for 54 patients; (**B**) non-bleeding controls on seven days—t1 and on twenty-eight days of DOAC therapy—t2, paired data for 47 patients; (**C**) non-bleeding controls at baseline—t0 and on twenty-eight days of DOAC therapy—t2, paired data for 47 patients; (**D**) bleeding cases at baseline—t0 and on seven days of DOAC therapy—t1; (**E**) bleeding cases on seven days—t1 and on twenty-eight days of DOAC therapy—t2; (**F**) bleeding cases at baseline—t0 and on twenty-eight days of DOAC therapy—t2; paired data for 16 patients in bleeding cases in (**D**–**F**)).

When patients were stratified after having experienced any bleeding event, no difference were noticed in *ADAMTS7* promoter methylation for any paired timepoint in non-bleeding patients (from t0 to t1: 15.6% vs. 15.0%, *p* = 0.337; from t1 to t2: 14.1% vs. 14.1%, *p* = 0.933; from t0 to t2: 14.5% vs. 14.2%, *p* = 0.561) (Figure 4A–C). In bleeding cases, *ADAMTS7* promoter methylation was significantly decreased from t0 to t2 (17.1% vs. 13.4%, *p* = 0.010). A gradient towards reduced *ADAMTS7* promoter methylation was noticed in bleeding cases from t0 to t1 (17.1% vs. 15.1%, *p* = 0.084) and from t1 to t2 (15.1% vs. 13.4%, *p* = 0.094); however, differences were not statistically significant (Figure 4D–F). Our study detected a statistically significant (*p* = 0.05) difference of 3.7% in *ADAMTS7* promoter methylation (4.9 standard deviation of the mean difference) in the 16 pairs of bleeding cases with 80.6% power.

Similar findings were obtained when patients were further stratified per drug category treatment. In bleeding cases only, *ADAMTS7* promoter methylation was significantly decreased from t0 to t2 irrespective of drug treatment (17.6% vs. 14.0%, *p* = 0.037 in factor Xa inhibitor treated patients, 14.7% vs. 10.8%, *p* = 0.053 in thrombin inhibitor treated patients).

To further assess whether it is bleeding that leads to *ADAMTS7* promoter demethylation or *ADAMTS7* promoter demethylation that increases bleeding probability, regression analysis was used. Logistic regression analysis with bleeding status as the dependent variable and *ADAMTS7* t0–t2 methylation difference, age, gender, hypertension, DOAC dose and renal function as contributing variables estimated that only hypertension increases bleeding odds (OR 19.22, 95% C.I. 1.78–207.64, *p* = 0.015), whereas *ADAMTS7* demethylation marginally decreases bleeding odds (OR 0.82, 95% C.I. 0.67–0.99, *p* = 0.043). By using linear regression adjusted for bleeding, age, gender, hypertension, DOAC dose and renal function, we found that bleeding was significantly associated with *ADAMTS7* promoter demethylation (β −3.125, 95% C.I. −6.208, −0.042, *p* = 0.047).

#### 3.3.4. Effect of Sex and Co-Morbidities on *ADAMTS7* Promoter Methylation

To assess whether sex affects baseline *ADAMTS7* promoter methylation, sex analyses were performed. Both in total population and in AF patients, no sex differences were present in *ADAMTS7* promoter methylation (total population: 15.8% in males vs. 15.5% in females, *p* = 0.861; AF patients: 15.0% in males vs. 16.1% in females, *p* = 0.517). Additionally, the effect of diabetes, hypertension and dyslipidemia on *ADAMTS7* promoter methylation was assessed in AF patients. The percentage of *ADAMTS7* promoter methylation did not differ in any subanalysis (diabetes vs. non-diabetes: 15.2% vs. 15.9, *p* = 0.722; hypertension vs. non-hypertension: 15.6% vs. 15.9%, *p* = 0.875; dyslipidemia vs. non-dyslipidemia: 17.1% vs. 14.1%, *p* = 0.080).

## 4. Discussion

In the present study, we analyzed how DOAC therapy or DOAC-related bleeding alter *ADAMTS7* promoter methylation over time in naïve AF patients. To the best of our knowledge, this is the seminal study assessing the potential of DOACs to induce epigenetic modifications. We have found that 28 days of DOAC therapy marginally decreased *ADAMTS7* promoter methylation from baseline in DOAC-treated AF patients and that this effect was statistically significant in patients who reported DOAC-related bleeding events. No other differences in *ADAMTS7* promoter methylation pattern were identified between AF patients and non-AF controls or within AF patients after being stratified per sex or co-morbidities.

DOAC treatment is the cornerstone of oral anticoagulant therapy in AF patients. Beyond their profound mechanism of action on the coagulation cascade through the direct inhibition of thrombin (dabigatran) or factor Xa (rivaroxaban, apixaban, and edoxaban), it is largely unknown whether DOACs alter the molecular signaling of coagulation cascade by inducing additional negative regulatory mechanisms on hemostasis. These mechanisms could include DOAC-induced epigenetic alterations in various genes and thus gene expression regulation of different enzymes with a role in coagulation, cardiovascular health, endothelial integrity and/or wound healing when a bleeding event occurs. ADAMTS7 is a well-known enzyme assessed as for its role in cardiovascular disease pathogenesis [21]. After taking into account that *ADAMTS7* substrates, namely COMPT, TSP-1 and TIMP-1, play a role in coagulation, as well as in vascular homeostasis, we have herein assessed the potential effect of DOAC therapy on *ADAMTS7* expression through the estimation of the percentage methylation of the *ADAMTS7* promoter.

The results of our study show that 28 days of DOAC treatment reduce the methylation of the *ADAMTS7* promoter in the pooled patient population (mean difference −1.3%, *p* = 0.044) and, more interestingly, that *ADAMTS7* demethylation is significant in patients reporting a DOAC-related minor bleeding event (mean difference −3.7%, *p* = 0.010). Regression analyses showed that *ADAMTS7* demethylation marginally decreased bleeding odds, whereas bleeding incidence was significantly associated with *ADAMTS7* promoter demethylation. We can thus conclude that it is bleeding incidence that leads to *ADAMTS7* promoter demethylation and *ADAMTS7* methylation signature is not a risk factor for bleeding.

Since this is the seminal study on the potential effect of DOACs and of DOAC-related bleeding on *ADAMTS7* methylation, no further comparisons can be made with the published literature. The potential explanation of our findings, however, may be mediated by the functional role of ADAMTS7 substrates. ADAMTS7 substrates are not yet well known; among these, the most well characterized are COMP, TSP-1 and TIMP-1, each of them having a role in the coagulation cascade, tissue remodeling and wound healing, indicating that increased ADAMTS7 expression could trigger different molecular pathways and drive substrate-associated effects on different biological systems [7,8,9,10,11,12].

The most studied ADAMTS7 substrate is COMP, an endogenous thrombin inhibitor. COMP is essential for maintaining vascular homeostasis and is considered to be a negative regulator of hemostasis and thrombosis [10,22,23]. Our initial hypothesis was that DOACs may increase ADAMTS7 promoter methylation and inactivate or reduce ADAMTS7 expression leading to decreased COMP degradation, increased COMP action as an endogenous thrombin inhibitor and a subsequent induction of an anticoagulable state. What we found instead was a marginally significant demethylation of *ADAMTS7* after 28 days of DOAC treatment. Additionally, *ADAMTS7* demethylation appeared significant only in factor Xa inhibitor-treated patients, whereas dabigatran treatment had no effect on *ADAMTS7* promoter methylation. A class effect is present between factor Xa inhibitors and the thrombin inhibitor that could be reflected in distinct molecular changes, and this should not be ignored.

Prompted by this finding, we have further stratified patients into bleeding cases and controls and, in this analysis, we have found that the reduction in *ADAMTS7* promoter methylation was significant only in bleeding cases, both before and after stratification in factor Xa inhibitors and thrombin inhibitor treatment. This result persisted both in factor Xa inhibitor- and in thrombin inhibitor-treated patients. It can thus be considered that it is the bleeding event occurrence that drives the result of *ADAMTS7* demethylation in the pooled AF patient cohort. It can be extrapolated that *ADAMTS7* demethylation in bleeding cases leads to increased ADAMTS7 expression, induced COMP cleavage and, subsequently, to an increase in thrombin and thrombin-induced platelet activation. This mechanism could counteract the overanticoagulant state of the patients in the case of an adverse bleeding event. Further speculations on the triggering effect of bleeding on *ADAMTS7* demethylation could also involve chemokines and chemokine-dependent prothrombotic processes [24]. ADAMTS7 expression has been found to be induced *in* *vitro* by cytokines such as tumor necrosis factor alpha (TNFa) and platelet-derived growth factor homodimer BB (PDGF-BB) [25], both known for their prothrombotic and procoagulant effects [26,27].

Other ADAMTS7 substrates which hold a role in angiogenesis, endothelium repair and would healing, when inactivated via increased ADAMTS7 expression, could also be associated with a bleeding-related activated mechanism. Among ADAMTS7 substrates, TSP-1 has been extensively studied for its key role in angiogenesis; TSP-1 acts as an anti-angiogenic factor [11,28]. TSP-1 inactivation could modulate angiogenesis in bleeding cases. Moreover, the MMPs interstitial collagenase (MMP-1), type IV and V collagenases (MMP-2 and MMP-9), and stromelysin (MMP-3) [29] play a role in vascular tissue remodeling during various biological processes such as angiogenesis, embryogenesis, morphogenesis and wound repair [30,31,32]. TIMP-1 degradation can lead to a reduced inhibitory effect on MMPs and increase the abundance of active MMPs that are involved in the healing progression of wounds, or have a positive effect on the healing of wounds.

Though the above mechanisms can support the findings of our study, meaning the demethylation of the *ADAMTS7* promoter in AF patients who experienced any bleeding event, our results should be interpreted with caution. We analyzed *ADAMTS7* promoter methylation in genomic DNA purified from whole blood and the timepoints of DNA isolation were specified in the miR-CRAFT study design at baseline and at 7 and 28 days of DOAC therapy [18]. Bleeding events recorded in the miR-CRAFT population have been reported on the 28-day follow-up visit; however, the exact time of onset cannot be estimated. DNA methylation is recognized as a major epigenetic mechanism that regulates gene expression and is subjected to alterations induced by both endogenous and exogenous stimuli [33]. The required time for a complete demethylation to occur, however, is largely unknown, and it cannot be excluded that a greater reduction in *ADAMTS7* promoter methylation could not have been captured in our study owing to the miR-CRAFT design. Furthermore, the bleedings reported in the study population were only minor and non-clinically significant. Nevertheless, these events can be considered significant in the study of underlying mechanisms.

We should also consider that currently only hypermethylated promoters (>80%) can be clearly distinguished by hypomethylated promoters (<10%) for their effect on the transcriptional silencing of related genes [34], and that aberrant DNA methylation is mainly studied in cancer biology [35,36,37]. In our study, the *ADAMTS7* promoter was partially methylated with a mean methylation percentage estimated at 16% in AF patients and controls, and an approximate difference of 4% (t0-t2) was statistically significant in bleeding AF patients. Whether this difference has a biological role in ADAMTS7 expression (or in that of other genes) remains to be elucidated.

Strengths of miR-CRAFT study mainly rely within the study design that is balanced for two critical confounders of epigenetic modifications, namely cancer and insulin treatment. Additionally, DNA methylation signatures may be impacted by blood processing and storing prior to DNA isolation. To avoid unexpected biases in DNA methylation analyses, all blood samples were kept at room temperature and genomic DNA was isolated within 72 h of blood drawing. In our study, other comorbidities did not affect the percentage of *ADAMTS7* promoter methylation and the patient population did not undergo any other changes in prescribed drugs. It can thus be expected that DOAC therapy and bleeding events are the sole variables that can affect *ADAMTS7* methylation.

The limitations of the study should also be discussed. Our study was adequately powered to detect the methylation difference between bleeding cases and controls, however, reducing sample size in sub-group analyses hinders firm conclusions to be drawn. Additionally, we have herein analyzed *ADAMTS7* methylation in genomic DNA isolated from whole blood leukocytes. This global methylation cannot be directly correlated with tissue-specific methylation alterations. Though we have used a verified qMSP method for methylation estimation, we cannot distinguish the exact CpGs that are demethylated. Moreover, despite the finding that *ADAMTS7* methylation is altered, the gene expression or protein levels either of ADAMTS7 or of its substrates have not been analyzed.

## 5. Conclusions

In conclusion, our study provides initial evidence of the effect of DOAC-related bleeding on *ADAMTS7* promoter methylation. The results suggest that a bleeding event can mechanistically induce *ADAMTS7* promoter demethylation, ADAMTS7 activation and subsequent hemostasis and endothelium repair via the degradation of ADAMTS7-dependent enzymes. However, additional studies in different ethnic groups are necessary to fully elucidate the effect of bleeding on ADAMTS7 expression and to further elucidate the ADAMTS7 substrates involved in such a process.

## Figures and Tables

**Table 1:** Demographic and clinical characteristics of patient population, stratified as bleeding cases and non-bleeding controls.

Characteristics	miR-CRAFT Population (*n* = 84)	Bleeding Cases (*n* = 16)	Non-Bleeding Controls (*n* = 68)
**Demographic**			
Male (*n*, %)	39 (46.4)	8 (50)	31 (45.6)
Age (years, mean ± SD)	69 (11)	71 (9)	69 (12)
Weight (kg, median, 25, 75 percentiles)	79 (65, 92)	72 (60, 92)	80 (65, 93.7)
Height (cm, median, 25, 75 percentiles)	170 (160, 176)	164 (156, 175)	170 (160, 177)
Smokers (*n*, %)	14 (16.7)	3 (18.7)	11 (16.2)
**Biochemical**			
Hemoglobin (gr%, median, 25, 75 percentiles)	13.9 (12.5, 15.1)	14.6 (13.2, 14.9)	13.7 (12.5, 15.2)
Platelets (100/μL, mean ± SD)	232 (69)	232 (64)	232 (71)
Urea (mg/dL, median, 25, 75 percentiles)	40 (33, 52.5)	41 (37.2, 56.7)	40 (32, 50.7)
Creatinine (mg/dL, median, 25, 75 percentiles)	0.94 (0.8, 1.1)	0.89 (0.77, 1.01)	1.00 (0.80, 1.20)
Creatinine Clearance (mL/min, median, 25, 75 percentiles)	78.8 (52.1, 97.8)	66.8 (50.9, 108.2)	80.1 (57.7, 97.8)
SGOT (U/L, median, 25, 75 percentiles)	22 (18, 30)	19 (17, 26)	22.5 (18, 32.2)
SGOT > 40 U/L (*n*, %)	9 (10.7)	1 (6.3)	8 (11.7)
SGPT (U/L, median, 25, 75 percentiles)	25 (19, 36)	25 (20, 30)	25 (19, 37)
SGPT > 56 U/L (*n*, %)	8 (9.5)	1 (6.3)	7 (10.3)
**Clinical**			
CHA_2_DS_2_-Vasc score (median, 25, 75 percentiles)	3 (2, 4)	3 (2, 4)	3 (1.25, 4)
HAS-BLED score (median, 25, 75 percentiles)	0 (0, 1)	0 (0, 1)	0.5 (0, 1)
**Renal function based on GFR (data for 58 patients)**			
Normal (*n*, %)	16 (27.6)	4 (25.0)	12 (17.6)
Mildly decreased (*n*, %)	26 (44.8)	5 (31.3)	21 (30.9)
Moderately decreased (*n*, %)	14 (24.1)	6 (37.5)	8 (11.8)
Severely decreased (*n*, %)	2 (3.4)	-	2 (2.9)
Failure (*n*, %)	-	-	-
**DOAC therapy**			
Rivaroxaban (*n*, %)	28 (33.3)	6 (37.5)	22 (32.4)
Apixaban (*n*, %)	36 (42.9)	7 (43.8)	29 (42.6)
Dabigatran (*n*, %)	20 (23.8)	3 (18.7)	17 (25.0)
Bleeding, Minor (*n*, %)	16 (19.0)	16	-
**Co-morbidities**			
Hypertension (*n*, %)	45 (53.6)	15 (93.7)	30 (44.1) *
Type 2 Diabetes (*n*, %)	19 (22.6)	7 (43.7)	12 (17.6)
Dyslipidemia (*n*, %)	37 (44.0)	12 (75.0)	25 (36.7) **
**Co-medications**			
Salicylates (*n*,%)	3 (3.6)	1 (6.3)	2 (3.0)
Antiplatelets (*n*,%)	-	-	-
NSAIDs (*n*,%)	-	-	-

* *p* = 0.004 between bleeding cases and non-bleeding controls. ** *p* = 0.037 between bleeding cases and non-bleeding controls.

## Data Availability

The original contributions presented in this study are included in the article. Further inquiries can be directed to the corresponding authors.

## Abbreviations

The following abbreviations are used in this paper:

ADAMTS7	A disintegrin and metalloproteinase with thrombospondin motifs 7
DOAC	Direct oral anticoagulant
COMP	The cartilage oligomeric protein
TSP-1	Thrombospondin-1
TIMP-1	Tissue inhibitor of metalloproteinases-1
AF	Atrial fibrillation
MMPs	Matrix metalloproteinases
GFR	Glomerular filtration rate
CI	Confidence intervals
TNFa	Tumor necrosis factor alpha
PDGF-BB	Platelet-derived growth factor homodimer BB

## References

[1] Atzemian N., Mohammed S., Di Venanzio L., Gorica E., Costantino S., Ruschitzka F., Paneni F. (2025). Cardiometabolic disease management: Influences from epigenetics. Epigenomics.

[2] Rose K.W.J., Taye N., Karoulias S.Z., Hubmacher D. (2021). Regulation of ADAMTS Proteases. Front. Mol. Biosci..

[3] Mead T.J., Apte S.S. (2018). ADAMTS proteins in human disorders. Matrix Biol..

[4] Santamaria S., de Groot R. (2020). ADAMTS proteases in cardiovascular physiology and disease. Open Biol..

[5] Colige A., Monseur C., Crawley J.T.B., Santamaria S., de Groot R. (2019). Proteomic discovery of substrates of the cardiovascular protease ADAMTS7. J. Biol. Chem..

[6] MacDonald B.T., Keshishian H., Mundorff C.C., Arduini A., Lai D., Bendinelli K., Popp N.R., Bhandary B., Clauser K.R., Specht H. (2022). TAILS Identifies Candidate Substrates and Biomarkers of ADAMTS7, a Therapeutic Protease Target in Coronary Artery Disease. Mol. Cell Proteom..

[7] Liu C.J., Kong W., Ilalov K., Yu S., Xu K., Prazak L., Fajardo M., Sehgal B., Di Cesare P.E. (2006). ADAMTS-7: A metalloproteinase that directly binds to and degrades cartilage oligomeric matrix protein. Faseb J..

[8] Kessler T., Zhang L., Liu Z., Yin X., Huang Y., Wang Y., Fu Y., Mayr M., Ge Q., Xu Q. (2015). ADAMTS-7 inhibits re-endothelialization of injured arteries and promotes vascular remodeling through cleavage of thrombospondin-1. Circulation.

[9] Sharifi M.A., Wierer M., Dang T.A., Milic J., Moggio A., Sachs N., von Scheidt M., Hinterdobler J., Müller P., Werner J. (2023). ADAMTS-7 Modulates Atherosclerotic Plaque Formation by Degradation of TIMP-1. Circ. Res..

[10] Liang Y., Fu Y., Qi R., Wang M., Yang N., He L., Yu F., Zhang J., Yun C.H., Wang X. (2015). Cartilage oligomeric matrix protein is a natural inhibitor of thrombin. Blood.

[11] Liu B., Yang H., Song Y.S., Sorenson C.M., Sheibani N. (2024). Thrombospondin-1 in vascular development, vascular function, and vascular disease. Semin. Cell Dev. Biol..

[12] Caley M.P., Martins V.L., O’Toole E.A. (2015). Metalloproteinases and Wound Healing. Adv Wound Care (New Rochelle).

[13] Wang X., Li C., Liang A., Peng Y., Sun J., Huang D., Xu K., Ye W. (2016). Regulation of a disintegrins and metalloproteinase with thrombospondin motifs 7 during inflammation in nucleus pulposus (NP) cells: Role of AP-1, Sp1 and NF-κB signaling. Inflamm. Res..

[14] Li L., Wang S., Wang M., Liu G., Yang Z., Wang L. (2023). miR-654-5p Suppresses Migration and Proliferation of Vascular Smooth Muscle Cells by Targeting ADAMTS-7. Cells Tissues Organs.

[15] Ren W., Liang L., Li Y., Wei F.Y., Mu N., Zhang L., He W., Cao Y., Xiong D., Li H. (2020). Upregulation of miR-423 improves autologous vein graft restenosis via targeting ADAMTS-7. Int. J. Mol. Med..

[16] Du Y., Gao C., Liu Z., Wang L., Liu B., He F., Zhang T., Wang Y., Wang X., Xu M. (2012). Upregulation of a disintegrin and metalloproteinase with thrombospondin motifs-7 by miR-29 repression mediates vascular smooth muscle calcification. Arter. Thromb. Vasc. Biol..

[17] Zhang L., Zhao F., Li C., Li H., Tang Q., Chen Y., Yao Y., Ding Z., Xu Y., Chen A. (2020). Hypomethylation of DNA promoter upregulates ADAMTS7 and contributes to HTR-8/SVneo and JEG-3 cells abnormalities in pre-eclampsia. Placenta.

[18] Ragia G., Thomopoulos T., Chalikias G., Trikas A., Tziakas D.N., Manolopoulos V.G. (2024). Circulating microRNAs and DNA Methylation as Regulators of Direct Oral Anticoagulant Response in Atrial Fibrillation and Key Elements for the Identification of the Mechanism of Action (miR-CRAFT): Study Design and Patient Enrolment. J. Pers. Med..

[19] Ragia G., Pallikarou M., Michou C., Thomopoulos T., Chalikias G., Trikas A., Tziakas D.N., Manolopoulos V.G. (2025). Direct oral anticoagulants do not affect miR-27a-3p expression, a regulator of coagulation cascade, in atrial fibrillation patients. J. Thromb. Thrombolysis.

[20] Lu L., Katsaros D., de la Longrais I.A., Sochirca O., Yu H. (2007). Hypermethylation of let-7a-3 in epithelial ovarian cancer is associated with low insulin-like growth factor-II expression and favorable prognosis. Cancer Res..

[21] Kessler T., Schunkert H. (2023). Targeting ADAMTS-7: A Vaccination Against Atherosclerosis-and Its Complications?. Circulation.

[22] Zhang L., Feng Q., Kong W. (2024). ECM Microenvironment in Vascular Homeostasis: New Targets for Atherosclerosis. Physiology.

[23] Fu Y., Kong W. (2017). Cartilage Oligomeric Matrix Protein: Matricellular and Matricrine Signaling in Cardiovascular Homeostasis and Disease. Curr. Vasc. Pharmacol..

[24] Leberzammer J., von Hundelshausen P. (2023). Chemokines, molecular drivers of thromboinflammation and immunothrombosis. Front. Immunol..

[25] Wang L., Zheng J., Bai X., Liu B., Liu C.J., Xu Q., Zhu Y., Wang N., Kong W., Wang X. (2009). ADAMTS-7 mediates vascular smooth muscle cell migration and neointima formation in balloon-injured rat arteries. Circ. Res..

[26] Bauer K.A., ten Cate H., Barzegar S., Spriggs D.R., Sherman M.L., Rosenberg R.D. (1989). Tumor necrosis factor infusions have a procoagulant effect on the hemostatic mechanism of humans. Blood.

[27] Ernofsson M., Siegbahn A. (1996). Platelet-derived growth factor-BB and monocyte chemotactic protein-1 induce human peripheral blood monocytes to express tissue factor. Thromb. Res..

[28] Lawler J. (2022). Counter regulation of tumor angiogenesis by vascular endothelial growth factor and thrombospondin-1. Semin. Cancer Biol..

[29] Cabral-Pacheco G.A., Garza-Veloz I., Castruita-De la Rosa C., Ramirez-Acuña J.M., Perez-Romero B.A., Guerrero-Rodriguez J.F., Martinez-Avila N., Martinez-Fierro M.L. (2020). The Roles of Matrix Metalloproteinases and Their Inhibitors in Human Diseases. Int. J. Mol. Sci..

[30] Krishnaswamy V.R., Mintz D., Sagi I. (2017). Matrix metalloproteinases: The sculptors of chronic cutaneous wounds. Biochim. Biophys. Acta Mol. Cell Res..

[31] Wang X., Khalil R.A. (2018). Matrix Metalloproteinases, Vascular Remodeling, and Vascular Disease. Adv. Pharmacol..

[32] Kandhwal M., Behl T., Singh S., Sharma N., Arora S., Bhatia S., Al-Harrasi A., Sachdeva M., Bungau S. (2022). Role of matrix metalloproteinase in wound healing. Am. J. Transl. Res..

[33] Moore L.D., Le T., Fan G. (2013). DNA methylation and its basic function. Neuropsychopharmacology.

[34] Kanaya T., Kyo S., Maida Y., Yatabe N., Tanaka M., Nakamura M., Inoue M. (2003). Frequent hypermethylation of MLH1 promoter in normal endometrium of patients with endometrial cancers. Oncogene.

[35] Chen C., Wang Z., Ding Y., Wang L., Wang S., Wang H., Qin Y. (2022). DNA Methylation: From Cancer Biology to Clinical Perspectives. Front. Biosci..

[36] Bhootra S., Jill N., Shanmugam G., Rakshit S., Sarkar K. (2023). DNA methylation and cancer: Transcriptional regulation, prognostic, and therapeutic perspective. Med. Oncol..

[37] Manolopoulos V.G., Ragia G. (2024). Fluoropyrimidine Toxicity: The Hidden Secrets of DPYD. Curr. Drug Metab..

